# Outcomes and Molecular Features of Brain Metastasis in Gastroesophageal Adenocarcinoma

**DOI:** 10.1001/jamanetworkopen.2022.28083

**Published:** 2022-08-24

**Authors:** Charlton Tsai, Bastien Nguyen, Anisha Luthra, Joanne F. Chou, Lara Feder, Laura H. Tang, Vivian E. Strong, Daniela Molena, David R. Jones, Daniel G. Coit, David H. Ilson, Geoffrey Y. Ku, Darren Cowzer, John Cadley, Marinela Capanu, Nikolaus Schultz, Kathryn Beal, Nelson S. Moss, Yelena Y. Janjigian, Steven B. Maron

**Affiliations:** 1 Department of Medicine, New York Presbyterian/Weill Cornell Medicine, New York, New York; 2 Department of Epidemiology and Biostatistics, Memorial Sloan Kettering Cancer Center, New York, New York; 3 Division of Solid Tumor Oncology, Memorial Sloan Kettering Cancer Center, New York, New York; 4 Department of Pathology, Memorial Sloan Kettering Cancer Center, New York, New York; 5 Department of Surgery, Memorial Sloan Kettering Cancer Center, New York, New York; 6 Department of Digital Informatics and Technology Solutions, Memorial Sloan Kettering Cancer Center, New York, New York; 7 Department of Radiation Oncology and Brain Metastasis Center, Memorial Sloan Kettering Cancer Center, New York, New York; 8 Department of Neurosurgery and Brain Metastasis Center, Memorial Sloan Kettering Cancer Center, New York, New York

## Abstract

**Importance:**

Brain metastasis (BrM) in gastroesophageal adenocarcinoma (GEA) is a rare and poorly understood phenomenon associated with poor prognosis.

**Objectives:**

To examine the clinical and genomic features of patients with BrM from GEA and evaluate factors associated with survival.

**Design, Setting, and Participants:**

In this single-institution retrospective cohort study, 68 patients with BrM from GEA diagnosed between January 1, 2008, and December 31, 2020, were identified via review of billing codes and imaging reports from the electronic medical record with follow-up through November 3, 2021. Genomic data were derived from the Memorial Sloan Kettering–Integrated Mutation Profiling of Actionable Cancer Targets clinical sequencing platform.

**Exposures:**

Treatment with BrM resection and/or radiotherapy.

**Main Outcomes and Measures:**

Overall survival after BrM diagnosis.

**Results:**

Sixty-eight patients (median age at diagnosis, 57.4 years [IQR, 49.8-66.4 years]; 59 [86.8%] male; 55 [85.9%] White) participated in the study. A total of 57 (83.8%) had primary tumors in the distal esophagus or gastroesophageal junction. Median time from initial diagnosis to BrM diagnosis was 16.9 months (IQR, 8.5-27.7 months). Median survival from BrM diagnosis was 8.7 months (95% CI, 5.5-11.5 months). Overall survival was 35% (95% CI, 25%-48%) at 1 year and 24% (95% CI, 16%-37%) at 2 years. In a multivariable analysis, an Eastern Cooperative Oncology Group performance status of 2 or greater (hazard ratio [HR], 4.66; 95% CI, 1.47-14.70; *P* = .009) and lack of surgical or radiotherapeutic intervention (HR, 7.71; 95% CI, 2.01-29.60; *P* = .003) were associated with increased risk of all-cause mortality, whereas 3 or more extracranial sites of disease (HR, 1.85; 95% CI, 0.64-5.29; *P* = .25) and 4 or more BrMs (HR, 2.15; 95% CI, 0.93-4.98; *P* = .07) were not statistically significant. A total of 31 patients (45.6%) had *ERBB2* (formerly *HER2* or *HER2/neu*)–positive tumors, and alterations in *ERBB2* were enriched in BrM relative to primary tumors (8 [47.1%] vs 7 [20.6%], *P* = .05), as were alterations in *PTPRT* (7 [41.2%] vs 4 [11.8%], *P* = .03).

**Conclusions and Relevance:**

This study suggests that that a notable proportion of patients with BrM from GEA achieve survival exceeding 1 and 2 years from BrM diagnosis, a more favorable prognosis than previously reported. Good performance status and treatment with combination surgery and radiotherapy were associated with the best outcomes. *ERBB2* positivity and amplification as well as *PTPRT* alterations were enriched in BrM tissue compared with primary tumors; therefore, further study should be pursued to identify whether these variables represent genomic risk factors for BrM development.

## Key Points

**Question:**

What clinical features are associated with survival in patients with brain metastasis from gastroesophageal adenocarcinoma?

**Findings:**

In this cohort study of 68 patients with brain metastasis from gastroesophageal adenocarcinoma, median survival was 8.7 months from diagnosis, with overall survival rates of 35% at 1 year and 24% at 2 years. Patients with Eastern Cooperative Oncology Group performance status of 2 or greater and who received no surgery or radiotherapy had significantly decreased survival in a multivariable analysis.

**Meaning:**

These findings suggest that modern treatment approaches are associated with improved prognosis for patients with brain metastasis from gastroesophageal adenocarcinoma.

## Teaser

This cohort study characterizes the clinical and genomic features of patients with brain metastasis from gastroesophageal adenocarcinoma and evaluates the associations of surgical resection and radiotherapy use with survival.

## Introduction

Brain metastasis (BrM) is a rare complication of gastroesophageal adenocarcinoma (GEA)^1,2,3,4,5^ and is associated with neurologic morbidity and poor prognosis, with median survival historically reported to be in the range of 2 to 6 months after BrM diagnosis.^1,2,5,6,7,8,9,10^ Clinical risk factors for BrM development are poorly understood, although associations have been found with higher N stage and other metastases (particularly liver, lung, and bone).^5^ In patients with surgically resected esophageal cancer, prior neoadjuvant therapy was also associated with the development of isolated BrM^4^; however, this development may be confounded by the use of neoadjuvant therapy in patients with worse prognosis. Several studies have noted enrichment of *ERBB2* (formerly *HER2* or *HER2/neu*) overexpression among patients with esophageal cancer with BrM.^11,12,13^

Brain metastasis treatment in GEA is complicated by the fact that most systemic chemotherapeutics in use have limited permeability across the blood brain barrier.^14^ Therefore, BrM treatment relies on surgical resection and radiotherapy.^15^ In the past, whole brain radiotherapy (WBRT) was used almost universally for BrM-directed radiotherapy. However, in the past 10 years, advances in stereotactic radiosurgery (SRS) have allowed for the delivery of higher radiation doses to more focal areas of disease, without the serious adverse effects associated with WBRT, most notably cognitive impairment.^16,17,18^ As a result, SRS has largely overtaken WBRT as the preferred modality of radiation therapy, with WBRT reserved for diffuse disease. Studies^16,19,20^ comparing the efficacy of SRS vs WBRT have found noninferiority in regard to overall survival, although the risk of subsequently developing new BrM was increased with SRS treatment alone. Surgical techniques, too, have evolved in recent years to allow for more precise preoperative planning and the preservation of normal brain parenchyma.^21^ Given the limited data available regarding risk factors for and treatment of BrM from GEA, we sought to characterize the clinical and genomic features of patients with BrM from GEA. In addition, we evaluated associations of surgical resection and radiotherapy use with survival.

## Methods

We examined patients with GEA diagnosed between January 1, 2008, and December 31, 2020, who were seen at Memorial Sloan Kettering Cancer Center (MSKCC) and who developed BrM. Of 3044 patients, a total of 68 patients were identified via review of billing codes^22^ on the electronic medical record or via a machine learning algorithm applied on imaging reports to identify BrM.^23^ The presence of BrM was confirmed via manual medical record review of imaging reports and/or clinician notes. Patients with only leptomeningeal disease (ie, no parenchymal metastases) were excluded. The end of the follow-up period was November 3, 2021. Institutional review board approval was obtained for data collection from the MSKCC. Written informed consent was obtained from all participants for genomic testing, and consent for clinical data use was waived by the institutional review board because of minimal risk. This study follows the Strengthening the Reporting of Observational Studies in Epidemiology (STROBE) reporting guideline for cohort studies.

Clinical data were abstracted from the electronic medical record into the MSKCC Esophagogastric Database by 4 data abstractors using a predefined REDCap (Research Electronic Data Capture) survey with random secondary validation. Data on patients’ self-identified race and ethnicity were collected to assess for a racially representative patient population; categories were derived from the United States census. Structured sites of metastatic disease were derived from the Memorial Sloan Kettering–Metastatic Events and Tropisms database,^22^ and imaging reports were verified against clinician notes and pathology reports. All abstractors received specialized training to minimize interrater variability. Abstractors were not blinded to the study hypothesis. Genetic sequencing data was collected from the Memorial Sloan Kettering–Integrated Mutation Profiling of Actionable Cancer Targets clinical sequencing cohort,^24^ which captures mutations, structural variants (fusions), and copy number variations in more than 341 cancer-associated genes. These sequencing data are publicly available on Cbioportal.^25,26,27^ Sample sites were manually verified via review of pathology reports. For genomic comparison between primary tumor and BrM samples, the earliest sequenced samples were used.

Disease and treatment characteristics were summarized using numbers and percentages for categorical variables and medians and IQRs for continuous variables. Overall survival (OS) was calculated from the date of BrM diagnosis. A univariate Cox proportional hazards regression model was used to examine the association of baseline covariates and OS. A multivariable Cox proportional hazards regression model was constructed by including covariates that were associated with OS from univariate analysis at a *P* < .10 level.

Fifty-one patients had sequencing of their primary tumor, BrM, or both for alterations (mutations, fusions, or copy number variants) in cancer-associated genes. Genes present at a frequency of at least 15% across all samples were compared between primary tumor and BrM samples using the χ^2^ test or Fisher exact test when subgroups had numbers less than 5. Because of the limited number of matched primary tumor and BrM samples (n = 10), patients who had both a sequenced primary tumor and BrM sample had their primary sample excluded from statistical analysis to satisfy the independent observations assumption for statistical comparison. All statistical analyses were performed with the R statistical software package, version 4.0.4 (R Foundation for Statistical Computing). All *P* values were based on 2-tailed statistical analysis, with *P* < .05 considered statistically significant.

## Results

A total of 68 patients (median age, 57.4 years [IQR, 49.8-66.4 years]; 59 [86.8%] male and 9 [13.2%] female; 55 [85.9%] White) were identified as having BrM. Fifty-seven patients (83.8%) had primary tumors located in the distal esophagus or gastroesophageal junction rather than the stomach (Table 1), and 31 patients (45.6%) had *ERBB2*-positive tumors (defined as an immunohistochemical classification of 3 or greater or an immunohistochemical classification of 2 or greater and fluorescence in situ hybridization positivity), which was higher than the 15% to 20% overall incidence of *ERBB2*-positive GEA tumors.^28,29^

**Table 1.** Patient Demographic and Brain Metastasis Characteristics

Characteristic	Finding^a^ (N = 68)
Age at diagnosis, median (IQR), y	57.4 (49.8-66.4)
Sex	
Male	59 (86.8)
Female	9 (13.2)
Race and ethnicity^b^ (n = 64)	
American Indian	1 (1.6)
Asian	4 (6.3)
Black	2 (3.1)
Hispanic/Latino	5 (7.8)
White	55 (85.9)
Primary tumor	
Esophageal-gastroesophageal junction	57 (83.8)
Gastric	11 (16.2)
*ERBB2* positive^c^	31 (45.6)
Stage at initial diagnosis (highest of clinical or pathologic)	
I	6 (8.8)
II	5 (7.4)
III	13 (19)
IV	44 (65)
Primary tumor resected	27 (39.7)
Age at BrM diagnosis, mean (IQR), y	58.5 (51.3-67.8)
BrM present at initial diagnosis	8 (11.8)
Time to BrM from initial diagnosis, median (IQR), mo	16.9 (8.5-27.7)
Time to BrM from stage IV diagnosis, median (IQR), mo	11.6 (0.4-20.6)
No. of BrMs	
1	27 (39.7)
2	6 (8.8)
3	9 (13.2)
≥4	26 (38.2)
Location of BrMs^b^	
Cerebellar	34 (50.0)
Frontal	32 (47.1)
Parietal	23 (33.8)
Occipital	23 (33.8)
Temporal	18 (26.5)
Brainstem	5 (7.4)
Other	5 (7.4)
Presenting symptom^b^	
Focal neurologic deficit	23 (33.8)
Headache	15 (22.1)
Altered mental status/confusion	6 (8.8)
Seizure	5 (7.4)
Asymptomatic	21 (30.9)
ECOG at BrM diagnosis (n = 60)	
0	8 (13.3)
1	41 (68.3)
2	10 (16.7)
3	1 (1.7)
No. of extracranial sites of active disease, median (IQR)^d^ (n = 63)	3 (1-4)
Pharmacologic treatment received any time before BrM diagnosis	57 (83.8)
Pharmacologic treatment received at time of BrM diagnosis^e^	34 (50.0)
BrM procedural treatment modality	
Surgery and SRS	26 (38.2)
Surgery and WBRT	1 (1.5)
SRS alone	23 (33.8)
WBRT alone	14 (20.6)
No directed treatment	4 (5.9)
Pharmacologic treatment started within 60 d after BrM	20 (29.4)

Abbreviations: BrM, brain metastasis; ECOG, Eastern Cooperative Oncology Group; SRS, stereotactic radiosurgery; WBRT, whole brain radiotherapy.

^a^ Data are presented as number (percentage) of patients unless otherwise indicated. Sample size is 68 unless otherwise specified. Note that because of incomplete clinical records, race and/or ethnicity was unknown in 4 patients, ECOG performance status was unknown in 8 patients, and 5 patients had no contemporaneous staging images available.

^b^ Patients could identify as multiple races and ethnicities and present with multiple BrM locations and symptoms.

^c^ Immunohistochemical classification of 3 or greater or an immunohistochemical classification of 2 or greater and fluorescence in situ hybridization positivity.

^d^ Extracranial sites of active disease (defined as number of individual organs affected) were assessed by staging images (whole body positron emission tomography/computed tomography or chest, abdomen, and pelvis computed tomography) performed within 60 days of BrM diagnosis, which could include the primary site.

^e^ Patients were considered to be receiving treatment at time of BrM if any treatment was received within 30 days before BrM diagnosis.

Most patients developed BrM late into their cancer course, with a median of 16.9 months (IQR, 8.5-27.7 months) from primary cancer diagnosis to BrM and 11.6 months (IQR, 0.4-20.6 months) from stage IV diagnosis to BrM (Table 1). Patients with *ERBB2-*positive disease did not have a substantial difference in median time to BrM (17.3 months [IQR, 1.6-27.6 months]) compared with those with *ERBB2-*negative disease (16.9 months [IQR, 11.5-27.4 months]). By the time of BrM diagnosis, 57 patients (83.8%) had received some form of pharmacologic therapy for GEA, and 27 (40.0%) had undergone resection of their primary tumor. A total of 34 patients (50.0%) were receiving systemic therapy at the time of BrM diagnosis.

Consistent with the long duration from initial diagnosis to BrM, many patients had active extracranial disease at the time of BrM diagnosis, with a median of 3 (IQR, 1-4) extracranial sites involved (assessed by contemporaneous staging computed tomography or positron emission tomography/computed tomography, with sites defined as number of individual organs affected). Although 27 patients (39.7%) presented with a solitary BrM at the time of BrM diagnosis, most patients presented with multiple, with 26 patients (38.2%) presenting with 4 or more. The most common locations for BrMs were in the cerebellum (34 [50.0%]) and frontal lobe (32 [47.1%]). A total of 47 patients (69.1%) were symptomatic at presentation, with focal neurologic deficit being the most common presentation (23 patients [33.8%]), but nearly one-third of patients (21 [30.9%]) were asymptomatic (Table 1), with incidental detection of BrM on staging positron emission tomography/computed tomography imaging or clinical trial screening brain magnetic resonance imaging. After diagnosis of BrM, most patients received BrM-directed therapy with radiotherapy alone (23 [33.8%] with SRS and 14 [20.6%] with WBRT) or combined surgical resection and radiotherapy (26 [38.2%] with SRS and 1 [1.5%] with WBRT). Twenty patients (29.4%) also began systemic therapy within 2 months of BrM diagnosis (Table 1).

**Figure 1.**
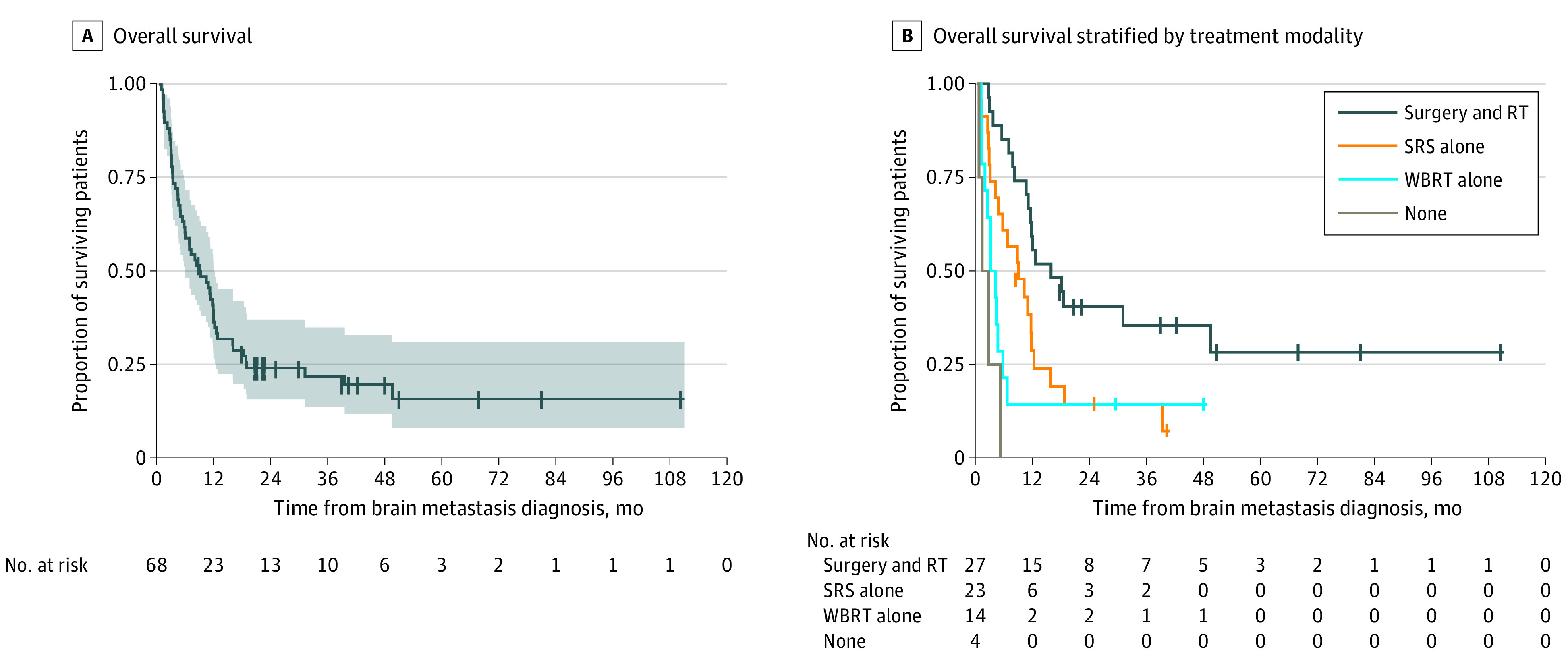
Kaplan-Meier Survival Curves Shaded area represents 95% CI. RT indicates radiotherapy; SRS, stereotactic radiosurgery; WBRT, whole brain radiotherapy.

Although the median survival from the time of BrM was only 8.7 months (95% CI, 5.5-11.5 months), 1-year OS was 35% (95% CI, 25%-48%) and 2-year OS was 24% (95% CI, 16%-37%) (Figure 1A). Univariate survival analysis demonstrated that Eastern Cooperative Oncology Group (ECOG) performance status of 2 or 3 vs 0 (hazard ratio [HR], 4.01; 95% CI, 1.35-11.9; *P* = .01), having 3 or more extracranial sites of disease vs 0 sites (HR, 2.89; 95% CI, 1.11-7.54; *P* = .03), having 4 or more BrMs (HR, 2.12; 95% CI, 1.14-3.93; *P* = .02), and treatment with surgery alone (HR, 2.16; 95% CI, 1.12-4.14; *P* = .02) or radiotherapy alone (HR, 3.61; 95% CI, 1.70-7.68; *P* < .001) vs surgery and radiotherapy combined were significantly associated with inferior survival from the time of BrM (Table 2). However, age and *ERBB2* status were not significantly associated with survival from time of BrM diagnosis. In a multivariable model (Table 2), an ECOG performance status of 2 or 3 was significantly associated with decreased survival (vs ECOG performance status of 0: HR, 4.66; 95% CI, 1.47-14.7; *P* = .009), and patients with an ECOG performance status of 1 also experienced a nearly 2-fold increased risk of death, although this increase was not significant (HR 1.79; 95% CI, 0.65-4.94; *P* = .26). Compared with patients treated with surgery plus radiotherapy, forgoing treatment of BrM was associated with significantly decreased survival after BrM diagnosis (HR, 7.71; 95% CI, 2.01-29.60, *P* = .003) (Figure 1B). Radiotherapy alone also resulted in a nearly 2-fold increased risk of death for those treated with SRS alone (HR, 1.92; 95% CI, 0.93-3.97; *P* = .08) or a 3-fold increased risk of death for those treated with WBRT alone (HR, 2.94; 95% CI, 0.95-9.06; *P* = .06) compared with surgery plus radiotherapy, but neither reached statistical significance. Neither the number of extracranial sites of disease nor the number of BrMs was significantly associated with OS in the multivariable model, although compared with patients with only 1 BrM, patients with 4 or more BrMs experienced at least a 2-fold increased risk of death (vs 1 BrM: HR, 2.15; 95% CI, 0.93-4.98; *P* = .07).

**Table 2.** Univariate and Multivariable Survival Analysis^a^

Characteristic	No. of patients	Univariate		Multivariable	
		Hazard ratio (95% CI)	*P* value	Hazard ratio (95% CI)	*P* value
Age	68	1.00 (0.98-1.02)	.80	NA	NA
*ERBB2* positivity	68	0.78 (0.46-1.35)	.38	NA	NA
ECOG PS at BrM diagnosis	60				
0	8	1 [Reference]	NA	1 [Reference]	NA
1	41	1.77 (0.69-4.55)	.23	1.79 (0.65-4.94)	.26
2-3	11	4.01 (1.35-11.9)	.01	4.66 (1.47-14.70)	.009
Extracranial sites of disease	63				
0	9	1 [Reference]	NA	1 [Reference]	NA
1-2	21	1.23 (0.45-3.37)	.68	1.13 (0.39-3.28)	.82
≥3	33	2.89 (1.11-7.54)	.03	1.85 (0.64-5.29)	.25
No. of brain metastases	68				
1	27	1 [Reference]	NA	1 [Reference]	NA
2-3	15	1.62 (0.79-3.32)	.19	1.48 (0.61-3.57)	.38
≥4	26	2.12 (1.14-3.93)	.02	2.15 (0.93-4.98)	.07
Treatment	68				
Surgery plus RT	27	1 [Reference]	NA	1 [Reference]	NA
SRS alone	23	2.16 (1.12-4.14)	.02	1.92 (0.93-3.97)	.08
WBRT alone	14	3.61 (1.70-7.68)	<.001	2.94 (0.95-9.06)	.06
None	4	11.7 (3.63-37.6)	<.001	7.71 (2.01-29.60)	.003

Abbreviations: BrM, brain metastasis; ECOG, Eastern Cooperative Oncology Group; NA, not applicable; PS, performance status; RT, radiotherapy; SRS, stereotactic radiosurgery; WBRT, whole brain radiotherapy.

^a^ Univariate and multivariable survival analysis using a Cox proportional hazards regression model demonstrating inferior survival in patients with worse PS, more BrMs, or absence of BrM procedural therapy. Age and *ERBB2* status were excluded in multivariable analysis as *P* > .10 in univariate analysis.

To explore potential genetic associations with BrMs, we examined the genetic landscape of BrMs compared with primary tumor tissue within our cohort. Among the 341 genes for which all samples were sequenced, the most commonly altered genes across all primary tumor and BrM samples were *TP53* (41 [80.4%]), *CDKN2A* (16 [31.4%]), *ERBB2* (15 [29.4%]), *SMAD4* (11 [21.6%]), and *PTRPT* (11 [21.6%]); alterations in *ARID1A* were present in 9 samples (17.6%), *EGFR* in 9 samples (17.6%), *ERBB3* in 8 samples (15.7%), *ERBB4* in 9 samples (17.6%), and *RARA* in 8 samples (15.7%). Among these 10 genes, BrM samples showed statistically significant enrichment in *PTPRT* alterations (7 [41.2%] in BrM vs 4 [11.8%] in primary, *P* = .03) and nonsignificant enrichment in *ERBB2* alterations (8 [47.1%] in BrM vs 7 [20.6%] in primary, *P* = .05) (Table 3).

**Table 3.** Primary vs Brain Metastasis Alteration Frequencies^a^

Gene	No. (%) of frequencies		*P* value^b^
	Primary (n = 34)	BrM (n = 17)	
*PTPRT*	4 (11.8)	7 (41.2)	.03
*ERBB2*	7 (20.6)	8 (47.1)	.05
*ARID1A*	4 (11.8)	5 (29.4)	.14
*CDKN2A*	12 (35.3)	4 (23.5)	.39
*RARA*	4 (11.8)	4 (23.5)	.42
*ERBB4*	5 (14.7)	4 (23.5)	.46
*TP53*	26 (76.5)	15 (88.2)	.46
*SMAD4*	6 (17.6)	5 (29.4)	.47
*ERBB3*	6 (17.6)	2 (11.8)	.70
*EGFR*	6 (17.6)	3 (17.6)	>.99

Abbreviation: BrM, brain metastasis.

^a^ Alteration frequencies in BrM samples compared with primary tumor samples (derived from patients with BrMs but without sequenced BrMs); each sample reflects a unique patient. Genes present in at least 15% of all samples were included. Alterations include mutations, fusions (none observed in this cohort), and copy number variants.

^b^ *P* values are derived from the Pearson χ^2^ test or Fisher exact test (when cell value is <5).

**Figure 2.**
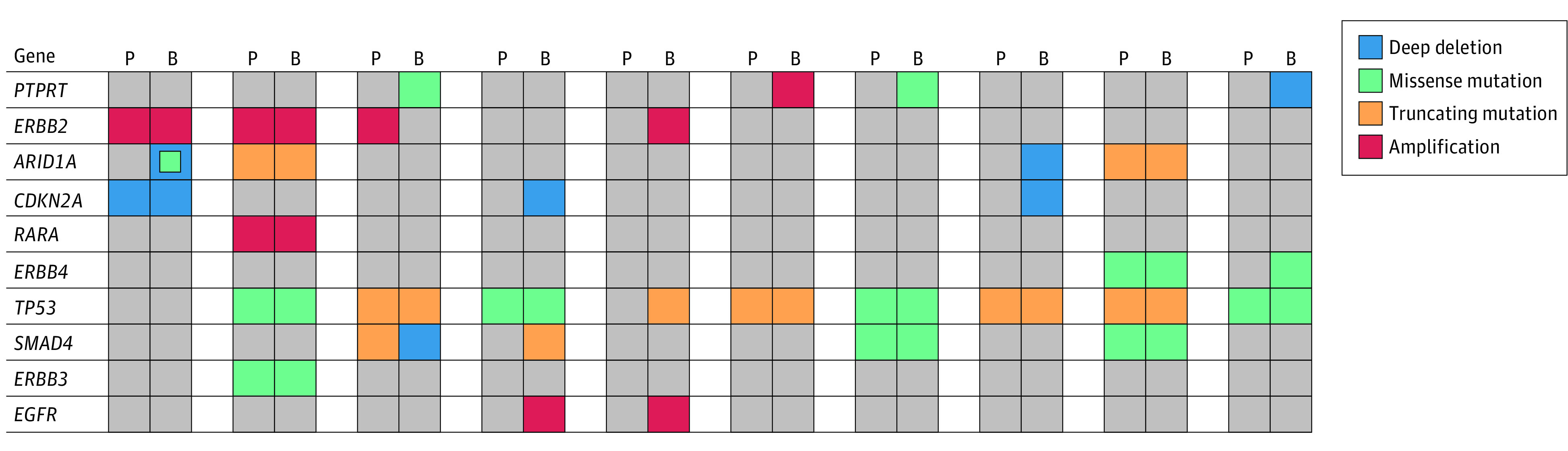
Oncoprints for 10 Patients With Matched Primary Tumor (P) and Brain Metastasis (B) Samples Each vertical bar represents 1 patient.

For the 10 patients with matched primary and BrM samples, we examined the alteration profile of the 10 genes altered in more than 15% of all samples (Figure 2). Alterations in *PTPRT* were varied, with 2 missense mutations, 1 amplification, and 1 deep deletion, all of which were not present in the primary tumor. *ERBB2* alterations were exclusively amplifications, which 1 patient acquired after initial primary sampling and 1 patient lost; 2 patients had persistent amplification. Acquired alterations in *ARID1A* (1 with deep deletion plus missense mutation and 1 with isolated deep deletion) and *CDKN2A* (2 with deep deletions) were also observed.

## Discussion

Brain metastasis remains a rare complication of GEA, perhaps related to the short survival seen in this disease overall, even in the modern era.^30,31,32,33^ The median survival from the time of BrM remained poor in this study (approximately 9 months), but this does reflect a longer survival than previously reported; however, as with prior studies,^1,2,6,7,8^ this was a limited case series. Remarkably, OS rates were 35% at 1 year and 24% at 2 years. This prolonged survival likely reflects a combination of case selection, advances in BrM surgical and radiotherapeutic techniques, and improvements in systemic therapies for extracranial disease, such as *ERBB2*-directed therapies and immune checkpoint inhibitors.^34^ Surprisingly, the presence of additional extracranial sites of disease was not significantly associated with a survival difference in a multivariable survival analysis. Although this outcome may be attributable to inadequate statistical power, the fact that significance was seen in the univariate analysis suggests that any survival differences seen with extracranial disease involvement were secondary to effects on performance status and/or BrM disease burden. As seen in prior studies,^7,8^ patients with poor ECOG performance status and numerous (≥4) BrMs tended to have decreased survival, although of the 2, only performance status was statistically significant on multivariable analysis.

In regard to the treatment of BrMs, the best outcomes were achieved with a combination of surgery and radiotherapy, which has control rates for index lesions on the order of 90% in modern case series.^35^ Of note, SRS alone resulted in a lower HR for death than WBRT alone, suggesting a benefit with modern radiation techniques.^16,19,20^ These survival benefits were seen even after accounting for ECOG performance status, number of brain metastases, and extracranial disease involvement, factors typically considered when selecting treatment modality.

As with prior studies,^11,12,13^ *ERBB2* expression and amplification were enriched in BrMs relative to the historically reported *ERBB2* positivity rate in GEA. *ERBB2* positivity, however, was not associated with a marked difference in time to BrM diagnosis (among patients with BrM) or survival from BrM diagnosis. Breast cancer data similarly identify enrichment of *ERBB2* alterations in BrM.^36,37,38^ It remains to be seen whether this enrichment is the result of selective tropism for the brain or a reflection of the central nervous system serving as a sanctuary site for disease, owing to the poor blood brain barrier penetration of *ERBB2*-directed therapies.^39^ We also identified significant enrichment in *PTPRT* alterations in BrM tissue. Of interest, in our analysis of matched BrM-primary samples, *PTPRT* alterations were always acquired (ie, not present in the primary tumor). Although its role in GEA is unknown, *PTPRT* is a tumor suppressor gene that is mutated in many solid tumors and when lost leads to increased *STAT3* expression.^40,41,42,43^ These mutations are thus associated with metastatic potential and chemotherapy resistance.^44,45,46^ Alterations in other phosphatases, including *PTEN* and the *PTPN* family, are similarly dysregulated in GEA.^47^ However, the impact of pathogenic *PTPRT* mutations in GEA requires further investigation. Finally, alterations in other GEA-associated genes^48,49,50,51,52^ were seen at high frequency in our cohort, including *TP53*, *EGFR*, *CDKN2A*, *SMAD4*, and *ARID1A*, but were not significantly enriched among BrM samples specifically. Of note, enrichment in *TP53* alterations has been observed in BrM from breast carcinoma, whereas enrichment in *EGFR* and *CDKN2A* alterations has been found in BrM from lung adenocarcinoma.^53,54^

### Limitations

Our study had several notable limitations. Because this was a retrospective study without a BrM-free matched population, we were unable to directly evaluate the association between specific genetic alterations and the risk of BrM development. Given the significant heterogeneity in the systemic treatments used and their timing compared with BrM development, we were also unable to evaluate the association of systemic therapy with survival in patients with BrMs. Furthermore, the heterogeneous timing of tissue collection and lack of tissue sampling in patients who did not receive surgical resection introduces selection bias. Finally, selection of fit patients with low systemic and central nervous system disease burden likely drove the remarkable benefit seen with combined surgery and radiotherapy.

## Conclusions

In this retrospective cohort study, we found that with modern-day surgical and radiotherapeutic management, a significant proportion of patients with BrM from GEA can achieve meaningful survival after BrM diagnosis. Good performance status and treatment with combination surgery and radiotherapy were associated with the best outcomes. We found that *ERBB2* positivity and amplification and *PTPRT* alteration were enriched in BrM tissue compared with primary tumors. Further study should explore whether these alterations represent genomic risk factors for BrM development to potentially identify and intervene on these lesions sooner.
